# Comparison of the virulence of *Streptococcus pneumoniae* in ICR mouse stocks of three different origins

**DOI:** 10.1186/s42826-019-0002-4

**Published:** 2019-06-24

**Authors:** Jun-Young Kim, Sun-Min Seo, Han-Kyul Lee, Han-Woong Kim, Yang-Kyu Choi

**Affiliations:** Department of Laboratory Animal Medicine, College of Veterinary Medicine, Konkuk University, 120 Neungdong-ro, Gwangjin-gu, Seoul, 05029 Republic of Korea

**Keywords:** *Streptococcus pneumonia* D39, Vancomycin, Survival rate

## Abstract

*Streptococcus pneumoniae* causes many people to suffer from pneumonia, septicemia, and other diseases worldwide. To identify the difference in susceptibility of and treatment efficacy against *S. pneumoniae* in three ICR mouse stocks (Korl:ICR, A:ICR, and B:ICR) with different origins, mice were infected with 2 × 10^6^, 2 × 10^7^, and 2 × 10^8^ CFU of *S. pneumoniae* D39 intratracheally. The survival of mice was observed until three weeks after the infection. The three stocks of mice showed no significant survival rate difference at 2 × 10^6^ and 2 × 10^7^ CFU. However, the lung and spleen weight in the A:ICR stock was significantly different from that in the other two stocks, whereas the liver weight in B:ICR stock was significantly lower than that in the other two stocks. Interestingly, no significant CFU difference in the organs was observed between the ICR stocks. The level of interferon gamma inducible protein 10 in Korl:ICR was significantly lower than that in the other two stocks. The level of granulocyte colony stimulating factor in B:ICR was significantly lower than in the other two stocks. However, tumor-necrosis factor-alpha and interleukin-6 levels showed no significant difference between the ICR stocks. In the vancomycin efficacy test after the *S. pneumoniae* infection, both the single-dose and double-dose vancomycin-treated groups showed a significantly better survival rate than the control group. There was no significant survival difference between the three stocks. These data showed that Korl:ICR, A:ICR, and B:ICR have no susceptibility difference to the *S. pneumoniae* D39 serotype 2.

## Introduction

*Streptococcus pneumonia* is a gram-positive pathogen that affects humans [1, 2]. The treatment for infection with *S. pneumoniae* requires taking antibiotics. This pathogen causes several infectious diseases such as pneumonia, meningitis [3], and acute otitis media [4] and acute sinusitis [5].

The occurrence of pneumococcal and acute respiratory infections is decreasing but is still considerably high in children under the age of 5 and in elders with weak immunity, in developing countries. Approximately 20–25% of all mortalities in children are identified to be a bacterial pneumonia caused by *S. pneumoniae* [4, 6]. The pathogen normally occupies the nasopharynx. The use of antibiotics in the treatment of the disease is impeded by the increase in the resistance of pneumonia strains to the therapy. Additionally, antibiotics cannot decrease the mortality rate of disease in the first 3 days of treatment, which emphasizes the need for deeper knowledge on the pathogenesis of pneumococcal disease [1]. Despite the high mortality worldwide, the host-pathogen interaction during *S. pneumoniae* infection is poorly understood and in depth knowledge is required [7].

The significance of animal models is well known especially in studying infectious diseases, as well as treatment efficacies. Animal models allow investigators to simulate various immune states and adjust the severity of infection in vivo, which allows in depth pharmacokinetic and pharmacodynamic measurements [8]. ICR mice are an outbred which Swiss in origin those are used in various field of research study. Multiple suppliers produce their own commercial ICR stocks which lead to characteristic varieties [9]. This study was performed to investigate the difference in the susceptibility of and treatment efficacy against *S. pneumoniae* serotype 2 D39 in differently originated ICR outbred mice.

## Materials and methods

### Animals

Seven-week-old male ICR mice were obtained from three difference sources. Korl:ICR was obtained from Koatech (Gyeonggi-do, Korea). A:ICR and B:ICR mice were purchased through Orient Bio Inc.(Gyeonggi-do, Korea) and Japan SLC (Shizuoka, Japan), respectively. Mice were acclimatized for a week at the pathogen-free animal facility at the College of Veterinary Medicine, Konkuk University (Seoul, Korea). Five mice were bred per sterilized polycarbonate cage and allowed 24-h access to sterilized food and water, and provided with sterilized wooden bedding. The facility was maintained with 12-h light/dark cycle and the temperature was maintained at 22 ± 2 °C and 50 ± 10% humidity. The surviving animals after the examination were euthanized in a CO_2_ gas chamber. All the procedures were approved by the Institutional Animal Care and Use Committee.

### *Streptococcus pneumoniae* D39 preparation

*S. pneumoniae* D39 serotype 2 was obtained from the Korean Centers for Disease Control and Prevention (KCDC, Osong, Korea) and stored at − 70 °C. The bacterial cells were placed at room temperature on ice until complete melting and cultured on a 5% sheep blood agar plate at 37 °C with 5% CO_2_ supplement for 16 h. Bacteria were harvested and re-suspended in sterilized phosphate buffered saline (PBS) to an optical density value of 0.3. Then the bacteria-PBS stock was transferred to brain-heart infusion broth (Merck, Darmstadt, Germany) and incubated at 37 °C with 5% CO_2_ for 6 h. The bacterial solution was centrifuged at 4500 rpm for 5 min. The concentration of bacteria was measured by optical density absorbance value at 600 nm using spectrometer. Colony forming units (CFU) per μL were counted using sheep blood agar plate with serially diluted bacterial suspension.

### Survival rate

*S. pneumoniae* D39 stocks were prepared at 2 × 10^6^, 2 × 10^7^, and 2 × 10^8^ CFU. Mice were intraperitoneally anesthetized with a mixture of Ketamine (Yuhan chemical Inc., Seoul, Korea) and Rumpun (Bayer Korea, Seoul, Korea). A total of 30 μL of *S. pneumoniae* suspension (2 × 10^6^, 2 × 10^7^, and 2 × 10^8^ CFU) was injected via exposed tracheae using a 31-gauge needle, via intratracheal injection. The skin incision was closed with suture materials. The clinical signs and death of ICR mice (*n* = 10 per group) were observed twice daily and body weight was measured once a day for 21 days after the injection of bacteria.

### Pathogenicity test

Three ICR stocks (n = 10 per group) were injected with 30 μL of 2 × 10^6^ CFU *S. pneumoniae* D39 intra-tracheally after anesthetization. The mice underwent autopsy at 48 h after the infection. Blood samples were collected via caudal vena cava and stored at − 4 °C overnight for serum separation. Liver, spleen, and lung were harvested and weighed in sterile conditions. Each organ was divided into two at each lobe; one half of the organ was immersed into liquid nitrogen and stored in a deep freezer (− 70 °C) and the other half was homogenized with sterile PBS for CFU analysis immediately after the extraction.

### Vancomycin efficacy test

Each ICR stock was injected with 30 μL of 2 × 10^6^ *S. pneumoniae* D39 intratracheally. The vancomycin single-dose treatment group (*n* = 6) of each ICR stock was treated with 30 mg/kg of vancomycin at 18 h after infection. Vancomycin double-dose treatment group (n = 6) was treated with 30 mg/kg of vancomycin at both 18 h and 42 h after infection. The control group (*n* = 10) was treated with PBS at both 18 h and 42 h after infection. The clinical signs and death of ICR mice were observed twice daily and body weight was measured once a day for 21 days after the injection of bacteria.

### CFU analysis

The liver, spleen, and lung tissues were weighed (30 mg) and homogenized with four times the titer of sterile PBS and subjected to serial dilution. The diluted homogenates were spread on 5% sheep blood agar plate and incubated for 24 h at 37 °C with 5% CO_2_. The *S. pneumoniae* colonies were then counted.

### Enzyme-linked immunosorbent assay (ELISA)

Blood serum was separated by centrifugation from the collected blood samples. The levels of the cytokines tumor necrosis factor-α (TNF-α), interleukin 6 (IL-6), interferon gamma inducible protein 10 (IP-10), and granulocyte colony stimulating factor (G-CSF) were measured using the Duoset ELISA kit (R&D systems, Minneapolis, MN) by following the manufacturers’ instruction. Blood serum was 1:5 in diluent buffer. The cytokine levels were analyzed using corresponding standard curve, and measurement of absorbance at 450 nm and 570 nm.

### Statistical analyses

Analyses of the data were performed through Prism 5 (Graphpad Software Inc.). Quantitative data are expressed as the mean values with standard deviation. Log-rank test was used to determine the survival difference and the significant difference between the groups was analyzed by the two-tailed Student’s *t*-test. The value of *P <* 0.05 was considered to be statistically significant.

## Results

### Survival rate examination in the different mouse stocks

**Fig. 1:**
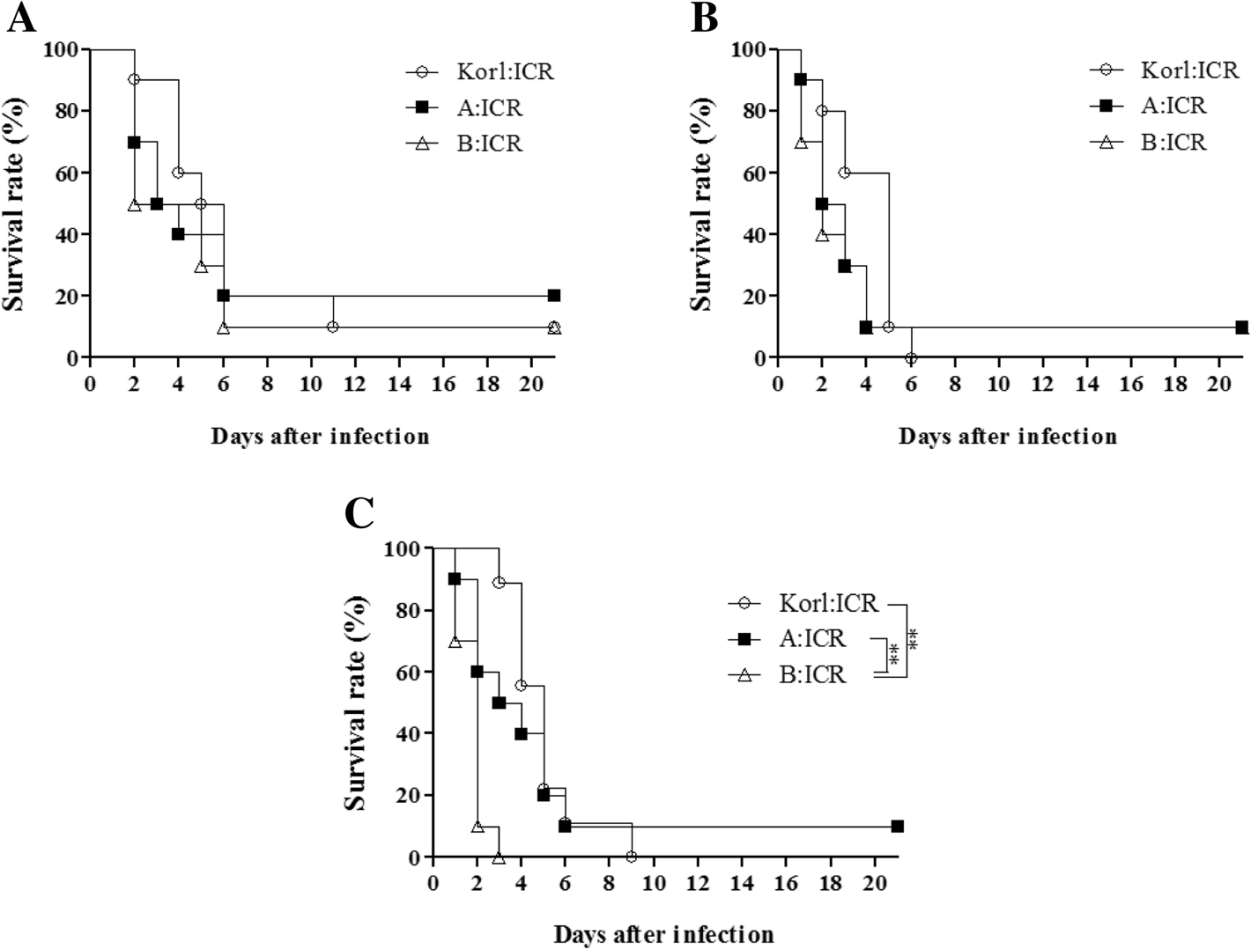
Survival rates (%) of Korl:ICR, A:ICR, and B:ICR stocks infected with *S. pneumoniae* D39 serotype 2 with CFU of 2 × 10^6^ (**a**), 2 × 10^7^ (**b**) and 2 × 10^8^ (**c**). Significant death rate of mice in B:ICR than in Korl:ICR and A:ICR was observed only at 2 × 10^8^ CFU (*P* < 0.01). *P* values were calculated by the log-rank test

To identify the survival difference among the three different mouse stocks, the survival rate was measured for 3 weeks after the *S. pneumoniae* D39 infection. When mice were infected with 2 × 10^6^ CFU bacteria, death in all the three stocks began at day 2 after the infection. The final survival percentage for Korl:ICR, A:ICR, and B:ICR was 10, 20, and 10%, respectively. No significant difference was observed between the stocks. When the mice were infected with 2 × 10^7^ CFU bacteria, the A:ICR and B:ICR groups exceeded 50% mortality at day 2 followed by Korl:ICR at day 5. None of the Korl:ICR mice survived, while 10% of A:ICR and B:ICR survived at the end of the experiment. No significant difference was observed between the stocks. When the mice were infected with 2 × 10^8^ CFU bacteria, 90% of the B:ICR mice died by day 2 after infection. Only 10% of the A:ICR mice survived till the end of experiment, while the other two stocks showed 0% survival. Significant difference was observed between Korl:ICR and B:ICR (*P* < 0.001), and A:ICR and B:ICR (*P* < 0.01) (Fig. 1).

### Pathogenicity examination in the different mouse stocks

**Fig. 2:**
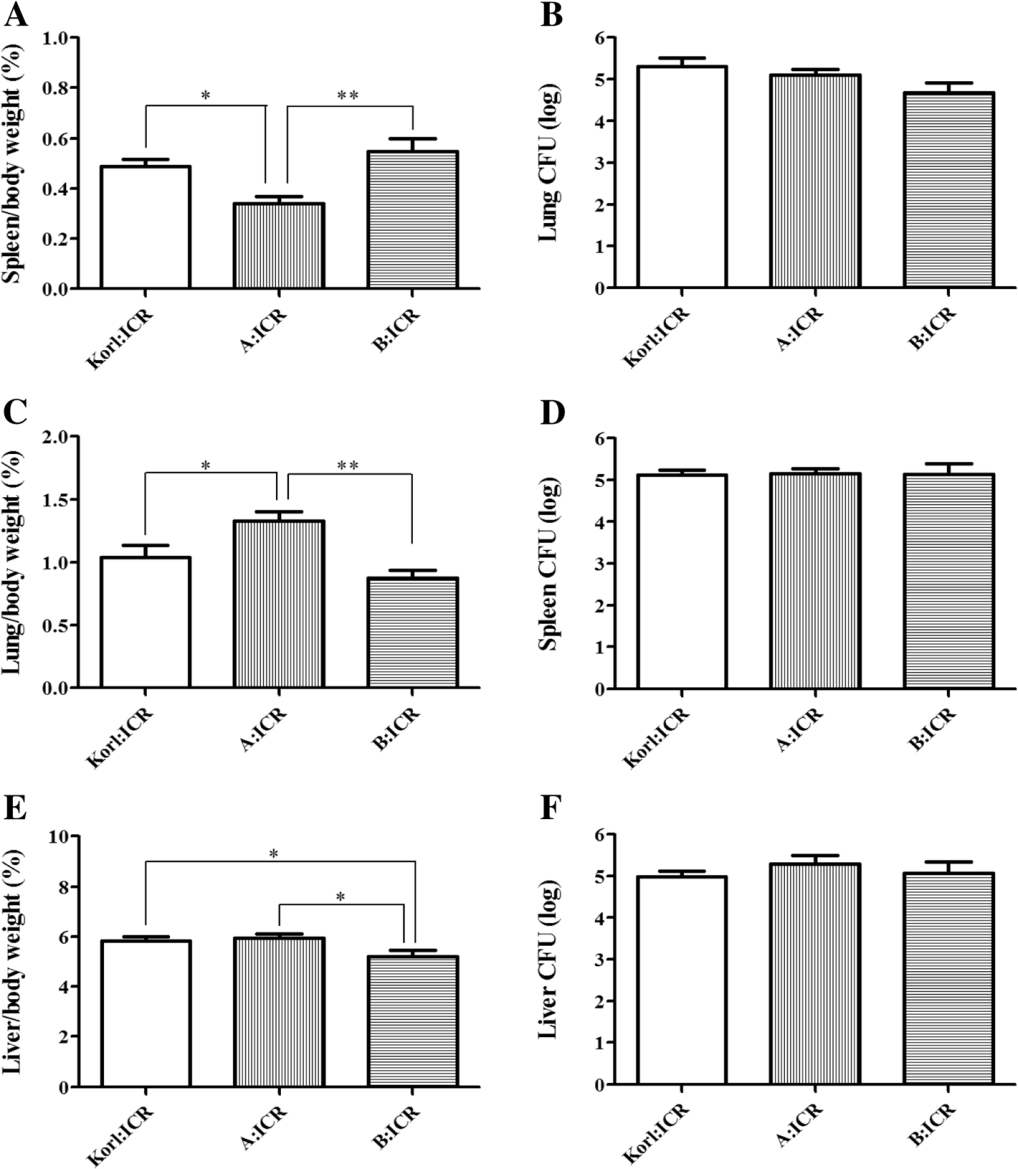
Weight of the lung (**a**), spleen (**c**), and liver (**e**) compared to the body weight (%) and CFU (log)/g of the lung (**b**), spleen (**d**), and liver (**f**) were measured 2 days after infection with 2 × 10^6^ CFU of *S. pneumoniae* D39 serotype 2. Lung and spleen weight in A:ICR were significantly different than in Korl:ICR and B:ICR. Liver weight in B:ICR was significantly lower than in Korl:ICR and A:ICR. Data are expressed as the mean ± SD. *P < 0.05, **P < 0.01 between the ICR stocks using two tailed *t-*tests

As shown in Fig. 2, the weight of each organ per body weight (%) varied among the different stocks. The lung weight in the A:ICR group was significantly heavier than in the other two stocks (*P* < 0.05) (Fig. 2a). The spleen weight was significantly lower in the A:ICR group (*P* < 0.05) (Fig. 2c) whereas the liver weight was significantly lower in the B:ICR group than in the other two stocks (*P* < 0.05) (Fig. 2e). On counting the bacterial numbers in each organ, more than 10^4^ CFU/g of *S. pneumoniae* D39 were calculated in the lung, spleen and liver of all three stocks. However, no significant difference was observed in the lung (Fig. 2b), spleen (Fig. 2d), and liver (Fig. 2f) between the groups. Hence, the organ per body weight did not influence the bacterial numbers to be greater or lesser in the organs.

**Fig. 3:**
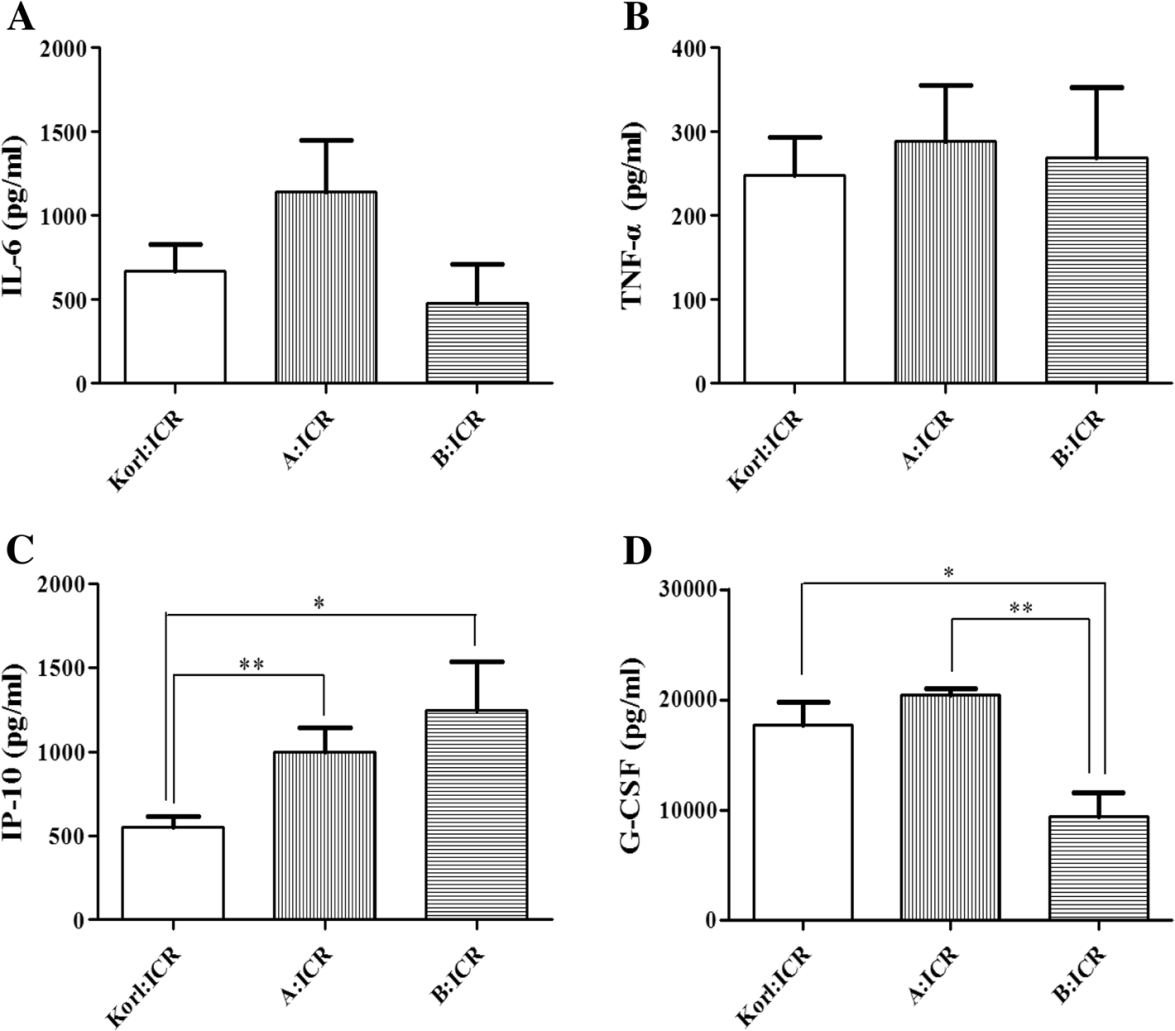
Serum cytokine levels of tumor necrosis factor-α (TNF-α) (**a**), interleukin 6 (IL-6) (**b**), interferon gamma inducible protein 10 (IP-10) (**c**), and granulocyte colony stimulating factor (G-CSF) (**d**) were measured 2 days after infection with 2 × 10^6^ CFU of *S. pneumoniae* D39 serotype 2. IP-10 level in Korl:ICR was significantly lower than in other ICR stocks. G-CSF level in B:ICR was significantly lower than in other ICR stocks. Data are expressed as the mean ± SD. *P < 0.05, **P < 0.01 between the ICR stocks using two tailed *t-*tests

TNF-α, IL-6, IP-10, and G-CSF cytokine levels were measured via ELISA using blood serum. The TNF-α and IL-6 levels were highest in the A:ICR group; however, no significant difference was observed between the stocks. The IP-10 level was significantly lower in the Korl:ICR group compared to the A:ICR (*P* < 0.05) and B:ICR (*P* < 0.01) groups. The serum G-CSF level was significantly lower in the B:ICR group than in the other two groups (*P* < 0.05) (Fig. 3).

### Effect of vancomycin treatment in the different mouse stocks

**Fig. 4:**
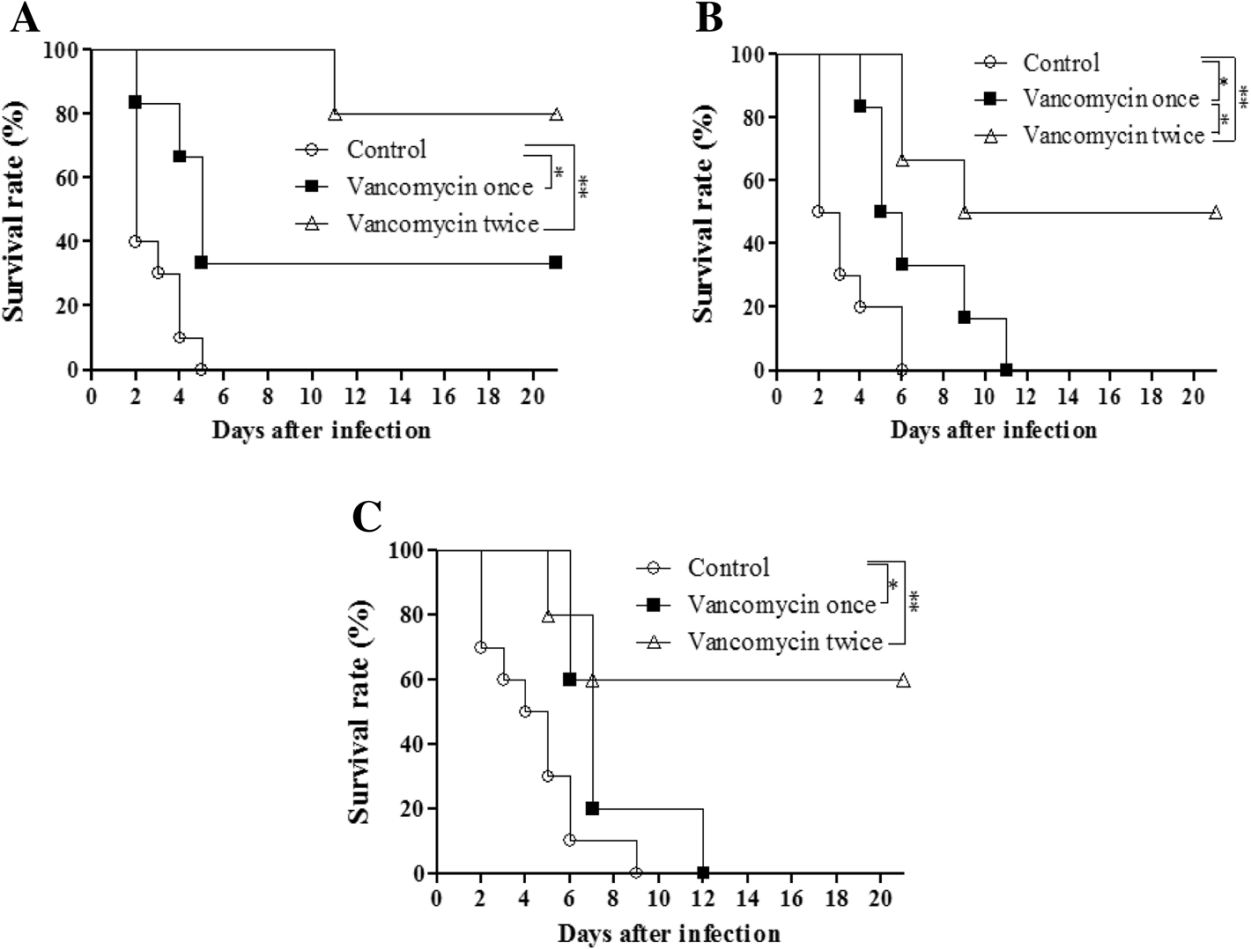
Survival rates (%) of Korl:ICR (**a**), A:ICR (**b**), and B:ICR (**c**) were observed after infection with 2 × 10^6^ CFU *S. pneumoniae* D39 serotype 2. These mice were treated with vancomycin single-dose or double-dose after 18 h, or 18 and 42 h. Treatment with vancomycin significantly improved the survival rate than that of the control mice in all the stocks (*P* < 0.05). Repetition of vancomycin injection enhanced the survival, while only A:ICR exhibited significantly improved survival rate in 18, 42 h than in the 18 h vancomycin treatment (*P* < 0.05). *P* values were calculated by the log-rank test. *P < 0.05, **P < 0.01 between the groups

To identify treatment susceptibility of the mouse stocks, 30 mg/kg of vancomycin was injected at 18 h or 18, 42 h after the bacterial infection. The death of mice in the control group began at 2 days after the infection and 100% mice died before the end of the experiment. However, the mice in the vancomycin double-dose group (18 h and 42 h after infection) were alive with 80, 50, and 60% surviving at the end of experiment in the Korl:ICR, A:ICR, and B:ICR group, respectively. In all the mouse stocks, the vancomycin single-dose and vancomycin double-dose treatment groups showed significantly better survival than the control group (*P* < 0.05). Furthermore, even though the double-dose treatment group had higher survival rate than the single-dose treatment group, significant (*P* < 0.05) difference was observed between these treatment groups in the A:ICR mice only (Fig. 4).

## Discussion

*S. pneumoniae* infection is fatal in children living in developing countries [10] and the entire population is at risk of suffering from pneumococcus infection throughout their life time [1]. Many studies use murine models to investigate the pneumococcal bacteria that cause pneumonia. This study was identified the susceptibility and treatment efficacy differences against *S. pneumoniae* serotype 2 D39 between Korl:ICR, A:ICR, and B:ICR sources.

A total of 2 × 10^6^, 2 × 10^7^, and 2 × 10^8^ CFU doses of *S. pneumoniae* were inoculated via intratracheal injection. The onset of death started 1 day after the infection with 2 × 10^7^ and 2 × 10^8^ CFU and from day 2 with 2 × 10^6^ CFU*.* The numbers of surviving mice at 21 days after infection with the CFU doses 2 × 10^6^, 2 × 10^7^ and 2 × 10^8^ were 4, 2, and 1, respectively. As shown in Fig. 1, the higher CFU value showed more rapid and vigorous death. Upon infection with 2 × 10^8^ CFU, the B:ICR mice showed a significantly higher death rate compared to the Korl:ICR and A:ICR stocks. In the other study, *S. pneumoniae* infection with 2 × 10^6^ CFU, also showed no significant susceptibility difference between C3H/HeN, C57BL/6, and ICR [11].

The A:ICR stock showed a significantly higher lung weight and lower spleen weight compared to the Korl:ICR and B:ICR mice (*P* < 0.05). However, the liver weight was significantly lower in the B:ICR mice. Usually, the enlargement of spleen caused by pathogen-induced hyperplasia [12] can be the hallmark of severity of a disease and the lung weight considerably increases in the case of severe pneumonia [13]. However, CFU analysis revealed no significant bacterial level differences in the spleen, lung, and liver between the three ICR stocks.

Cytokines such as tumor necrosis factor-alpha (TNF-α) and interleukin-6 (IL-6) are found at high concentration upon tissue injury or bacteria-induced organ damage [14], and has a role in bacterial clearance in pulmonary infection [15, 16]. In addition, TNF-α reduced the thymocyte apoptosis against *S. pneumonia* infection [17]. When BALB/c and C57BL/6 mice were infected with the *S. pneumoniae* serotype 3 WU2, spleen cytokine levels showed no alteration whereas infection with the *S. pneumoniae* serotype 14 DW14 in the same mice strains showed alteration of cytokine levels [18]. The pro-inflammatory cytokine IP-10 level was elevated by *S. pneumoniae* and *Neisseria meningitides* [19]. In 2 × 10^8^ CFU *S. pneumoniae* infection, survival rate of B:ICR was also significantly lower than the other two stocks (Fig. 1c). G-CSF assists neutrophils to be released into blood from bone marrow [20] and mice with deficient G-CSF receptor were highly sensitive to fungal and bacterial infection [21]. In case of mice infected with 2 × 10^6^ CFU of *S. pneumoniae,* high level of serum G-CSF after 2 days of infection in Korl:ICR and A:ICR mice was closely associated with an increase of survival rate (Figs. 1a and 3d). These results are similar to previous reports that G-CSF pretreatment in mice and rats increased the survival rate in *S. pneumoniae* infection [22, 23]. TNF-α and IL-6 levels were not significantly different between the three ICR stocks, while Korl:ICR showed a significantly lower IP-10 level than the other two ICR stocks. G-CSF level in B:ICR was significantly lower than the other two stocks.

Vancomycin is a glycopeptide antibiotic that inhibits gram-positive bacteria, and leads to decrease in IL-17-producing T-helper cells (Th17), which are potent inflammatory activators [24]. A vancomycin single-dose of 30 mg/kg and double-dose treatment after bacterial injection significantly increased the survival rate compared to that in the control group in all the ICR stocks. However, only the A:ICR stock showed a significantly better survival rate between the single-dose and double-dose vancomycin treatments. The A:ICR and B:ICR stocks treated with single-dose vancomycin showed 100% mortality; however, Korl:ICR showed 70% mortality. Thirty-three percent survived in Korl:ICR but no significant survival rate difference between the stocks was observed. The double-dose vancomycin treatment showed 80, 50, and 70% survival rate in Korl:ICR, A:ICR, and B:ICR, respectively. We found that the Korl:ICR stock was more sensitive to the antibiotic than the other ICR stocks. Repeated antibiotic treatment enhanced the survival rate of the three different stocks. Further study is needed to increase the survival rate beyond that obtained in the current study. Based on our results, due to the characteristics of the outbred ICR along with their different origins, the relative weight of the organs and the cytokine levels may vary, but the severity of infection caused by *S. pneumoniae* serotype 2 D39 was not significantly affected, except at high dose of bacterial load. Moreover, the repeated treatment with antibiotics increased the survival rate. Taken together, the Korl:ICR, A:ICR and B:ICR stocks have no difference in susceptibility to the *S. pneumoniae* D39 serotype 2 and this response profile may help other researchers searching for an ICR animal model in the future.

## Conclusion

Three ICR mice stocks (Korl:ICR, A:ICR, and B:ICR) of mice showed no significant survival rate difference when mice were infected with at 2 × 10^6^ and 2 × 10^7^ CFU. And TNF-α and IL-6 levels showed no significant difference between the ICR stocks. However, Korl:ICR mice showed a significantly lower IP-10 level while B:ICR mice showed a significantly lower G-CSF level than the other two stocks. Our results indicate that there is no susceptibility difference to *S. pneumonia* D39 serotype 2 upon three different ICR stocks.

## Abbreviations

CFU: Colony forming unit
ELISA: Enzyme linked immunosorbent assay
G-CSF: Granulocyte colony stimulating factor
IL-6: Interleukin 6
IP-10: Interferon gamma inducible protein 10
PBS: Phosphate buffered saline
*S. pneumoniae*: *Streptococcus pneumonia*
Th17: IL-17 producing T-helper cell
TNF- α: Tumor necrosis factor- α
